# Towards a Multi-Layered Phishing Detection

**DOI:** 10.3390/s20164540

**Published:** 2020-08-13

**Authors:** Kieran Rendall, Antonia Nisioti, Alexios Mylonas

**Affiliations:** 1 Department of Computing and Informatics, Bournemouth University, Bournemouth BH12 5BB, UK; s4908752@bournemouth.ac.uk; 2 Department of Computing and Mathematical Sciences, University of Greenwich, London SE10 9BD, UK; a.nisioti@greenwich.ac.uk

**Keywords:** supervised machine learning, phishing, multi-layer

## Abstract

Phishing is one of the most common threats that users face while browsing the web. In the current threat landscape, a targeted phishing attack (i.e., spear phishing) often constitutes the first action of a threat actor during an intrusion campaign. To tackle this threat, many data-driven approaches have been proposed, which mostly rely on the use of supervised machine learning under a single-layer approach. However, such approaches are resource-demanding and, thus, their deployment in production environments is infeasible. Moreover, most previous works utilise a feature set that can be easily tampered with by adversaries. In this paper, we investigate the use of a multi-layered detection framework in which a potential phishing domain is classified multiple times by models using different feature sets. In our work, an additional classification takes place only when the initial one scores below a predefined confidence level, which is set by the system owner. We demonstrate our approach by implementing a two-layered detection system, which uses supervised machine learning to identify phishing attacks. We evaluate our system with a dataset consisting of active phishing attacks and find that its performance is comparable to the state of the art.

## 1. Introduction

In recent years, phishing attacks have been on the rise and inevitably have caught the attention of the public. Phishing is a technique that is commonly used to obtain confidential information through acts of impersonation. In the current threat landscape, phishing constitutes most of the time the initial action [1] of any sophisticated attack [2]. Moreover, according to [3], 42% of business emails were compromised from phishing attacks since June 2018.

The more the popularity of personal IoT (Internet of Things) devices, such as mobiles, wearables, and smart assistants increases, the more these devices are used as phishing vectors by threat actors. The built-in functionality of these devices enables the opening of emails, websites, and the downloading of applications. Unfortunately, the protection that these devices offer to the end-users is rather limited [4,5]. Moreover, the authors in [6] reported that phishing can be conducted by exploiting the Android Instant App feature, thus bypassing the full installation process.

In 2020, phishing has been contextualised by exploiting a significant fear factor of the public. The general public has been targeted by large-scale phishing campaigns amid the COVID-19 pandemic, where threat actors were posing as government and other health authorities, such as the Center for Disease Control (CDC, Atlanta, GA, USA) and the World Health Organisation (WHO). At the same time, WHO employees were victims of targeted phishing attacks in an attempt to steal confidential information [7]. For instance, a malicious site was observed on 13 March 2020 impersonating WHO’s internal email system and harvesting logon credentials [8].

Regulations such as HIPPA and the more recent GDPR provide coherence around confidential data and at the same time bring a significant financial impact to businesses following a breach. In July 2019 alone, British Airways were fined £183M and Marriot Hotels £99M [9] upon assaults using phishing.

While security organisations, such as Norton and UK’s National Cyber Security Centre, are proactively advising the public on how to protect from this threat, a complementary approach for tackling it is needed. Currently, blacklisting is the most commonly used security control for detecting known phishing campaigns, i.e., those that are included in the blacklist. However, the short life cycle of phishing domains renders blacklisting ineffective, as the process of identifying, reporting, and blacklist updating is time-consuming. Specifically, [10] reported that 84% of phishing sites exist for less than 24 h, and [11] identified the life cycle of a phishing domain to be less than a benign domain.

For these reasons, the literature has explored data-driven approaches that aim to dynamically detect phishing domains using supervised or unsupervised machine learning [12]. However, the majority of approaches rely on single-layered models for detection, such as [13,14,15]. Their feature selection, in conjunction with their implementations, is focused mainly on the extraction of domain characteristics, such as URL length and the number of special characters, which could be easily tampered with by threat actors [16]. Similarly, many approaches have impractical feature extraction strategies, as they require large amounts of computational processing resources and may be constrained by TLS/SSL encryption [14,17]. Thus, such single-layered approaches are bounded by their high computational requirements, which does not allow them to be deployed in a real production environment. Despite the features that have a high computational overhead, by having a multilayered system, the expense of such features can be prioritised for uncertain classifications, and thus, computer power is not wasted unnecessarily.

In this paper, we propose a two-layered detection framework that uses supervised machine learning in order to identify phishing attacks. In our work, resource-demanding operations of the second layer can be avoided based on the prediction confidence of the first inexpensive detection layer. As a result, this saves infrastructure resources, which is a critical factor in the deployment of any security control to a production environment. At the same time, it achieves comparable accuracy with the past literature in the domain, which focuses on single-layer supervised machine learning.

In summary, this paper makes the following contributions:

- We propose a framework to detect active phishing attacks. The framework follows a two-layered approach to identify phishing domains based on supervised machine learning. We implement and evaluate the framework on a dataset that we created based on 5995 phishing and 7053 benign domains.
- We suggest and utilise features in each layer of the framework that have been used in the literature as well as propose a new feature for layer two. We discuss their ability to resist tampering from a threat actor who is trying to circumvent the classifiers, e.g., by typosquatting.

The rest of this paper is structured as follows: in Section 2, we discuss the background relevant to our framework, while Section 3 presents the related work. Section 4 presents our methodology and Section 5 our implementation. In Section 6, we present the evaluation setup and experimental results. Finally, Section 7 concludes the paper and discusses future work.

## 2. Background

### 2.1. Detection Methods

Any detection system has three fundamental components: a data collection sensor, pre-processing data functions, and a decision engine [18]. A sensor can either retrieve or be given data in the form of host data and/or network traffic. Nowadays, a popular decision-making technique for detection is using a signature/rule-based engine. The disadvantage of such a method is that it requires existing knowledge of the malicious activity in the form of predefined rules. To overcome this, Monzer et al. [19] created a model-based rule generation algorithm to minimise the manual effort of crafting a rule/signature. Despite signature-based detections being limited to detecting explicit patterns, they are powerful if one knows what to discover. Nevertheless, such methods are constrained by their rule requirement, which conflicts with the short life cycle of threat intelligence.

On the other hand, advanced statistical methods, such as supervised and unsupervised machine learning (ML) systems that have been widely deployed for various detection tasks do not have this disadvantage [20,21,22,23,24,25,26]. It is important to note that the objective of a detection system is to detect specific categories of activity while keeping false-positive (FP) and false-negative (FN) numbers low. Supervised techniques do not utilise rules but instead require training on past data in order to learn to classify new observations in predefined categories. However, one needs to be careful when training such a model, as underfitting or overfitting it on the training dataset negatively impacts performance. The other main factor, apart from the supervised algorithm in use, that affects the performance of the detection system is the chosen feature set. A higher number of features does not always equal better performance, while an optimal set of features can allow a model to cope with noise in data, seasonality, and trends.

This work considers four supervised machine learning algorithms, which have extensively been used for phishing detection [12], namely: Multilayer Perceptron, Support Vector Machine, Naïve Bayes, and Decision Trees.

Multilayer Perceptron (MLP) is a class of artificial neural network which has been widely used as a supervised machine learning algorithm to achieve a binary-classification output. During the training process, MLP weights and biases are adjusted to minimise error using the backpropagation technique. Then, they are passed to an activation function, which in this work is a hyperbolic tangent. Finally, to improve the model coefficients during learning and further reduce error, a parameter optimisation algorithm was used, i.e., the scaled conjugate gradient.

Support Vector Machine (SVM) classifies by fitting a hyperplane to the data that maximises the separability between the two classes and uses it as a decision boundary. A radial kernel function is used to handle the non-linear separability on the features.

Naïve Byes (NB) is a supervised machine learning algorithm that classifies using a probabilistic approach, i.e., the Bayes Theorem. The algorithm requires that features used are subject to independence and it can effectively model relatively small datasets.

The decision tree (DT) algorithm is a supervised machine learning algorithm that can be used in classification or regression problems. The algorithm is representative of a tree-like structure, with branches and lead nodes growing arbitrarily. As with most machine learning algorithms, a balanced dataset is important to reduce the likelihood of overfitting, i.e., lack data generalisation.

### 2.2. JDL Model

The Joint Directorate of Laboratories model (JDL) is a common framework for enabling situational awareness for decision support contexts. Initially developed for military systems, its use in large-scale critical infrastructure monitoring has been an effective framework to drive a structured and scalable information fusion system that incorporates the user with machine-driven analytics [27]. With respect to cyber defence, a lack of literature has been delivered applying the JDL framework. The reasons for this may vary, including a limited understanding across the levels of JDL [28].

## 3. Related Work

Malicious URLs constitute a vector for the realisation of different attacks, such as malware installation using a Domain Generated Algorithm for command and control [29] or for luring users as part of a phishing campaign. Existing detection and prevention techniques typically rely on blacklisting and supervised machine learning [30,31].

Blacklisting, which is the first line of defence against malicious websites, is today enabled by default in all popular browsers (i.e., Chrome, Firefox, Opera, and Safari) irrespective of the platform that they execute (desktop or mobile device). Browsers provide to their users different blacklisting technologies, such as Google Safe browsing and SmartScreen, with Google’s blacklist being the most prevalent. However, past work has proven that the protection that blacklists, as a security control, offer to their users is rather limited [4,5].

According to Bell and Komisarczuk [32], PhishTank and OpenPhish blacklists see up to a 24-h delay before updating, providing to threat actors a considerable window of opportunity. Therefore, relying on blacklisting can increase the risk exposure if an organisation is subject to a targeted phishing campaign. Moreover, threat actors do take advantage of world events or crises, as [33] has reported that Gmail service was blocking 18M Coronavirus scams per day during the Coronavirus pandemic. As a result, if one needs to keep up with phishing campaigns, then it is important to use data-driven approaches instead of solely relying on static knowledge provided by blacklists. Supervised machine learning methods, particularly those applied for URL classification [12] 15have been successful in the past in determining if the class of URL is malicious or benign.

Authors in [14] explored an approach to detect phishing which uses visual similarity signatures. While the dataset endured discrimination using characteristics in page styling, SSL certificates, and webpage contents, it required a large amount of processing. This can create computational overhead, which can reduce the applicability of the detection system in production environments. Hara et al. [17] also used image similarity, achieving a detection rate of 82.6% with a false-positive rate of 18%. They compared their approach to browser-based detection with Google Safe Browsing, which detected only 30.5%.

Other phishing detection approaches that have been used in the literature include applying associative rule-based mining algorithms on the URL. The work by Jeeva and Rajsingh [16] used the Apriori algorithm to identify the significance of a feature set. It was noted that the URL length and special characters were useful in detecting malicious domains and that most of the malicious sites were not using HTTPS. In addition, [34] reported that in 2016, less than 3% of phishing sites used HTTPS, rising to 33% in 2017. The increase over the years will likely reduce the effectiveness of identifying phishing using the previously mentioned strategies. Aburrous et al. [35] also used rule-based mining and identified URL and domain features as significant to determine if a website is malicious or benign. Abdelhamid et al. [36] identified features that correlate with the class of domain, i.e., malicious or benign, which included the age of the domain, URL length, HTTP/S, and others.

Another attempt to detect phishing more accurately was conducted in [37]. The approach used real Internet Service Provider traffic flows and modelled the data using a Deep Belief Network. The feature set involved static features seen in previous studies, but also interactive features that are less likely to be manipulated by threat actors. This included graphing IP addresses and calculating the in-degree and out-degree of the active URL’s. Although identifying interactive characteristics at an ISP level would be impractical due to resource constraints, Ma et al. [17] modelled a combination of other less-intrusive interactive features. These included publicly available WHOIS properties, such as domain creation data, registrar and Time-to-Live (TTL). However, Blum et al. [38] highlighted that using WHOIS data could create a bottleneck in detection speed. Moreover, Aaron and Rasmussen [39] reported that out of 255,065 phishing attacks, 90% of domains were not used on the same day as registration, and around 15–20% of domains would remain inactive for 1–12 months. This suggests the creation date as a feature could, in the future, be ineffective in discriminating significant proportions of phishing campaigns.

Stergiopoulos et al. [23] focused on overcoming the challenge that encryption carries in preventing certain detection techniques, such as URL extraction. To this end, a range of features was extracted at the packet level; more specifically, stemming from the time difference between packets and packet sizes. In the context of phishing, when a user accesses a site, a DNS request is sent to a DNS server to retrieve the respective IP address. As our results suggest, the response time for retrieving a DNS response relative to the DNS request could be indicative of an unpopular domain. For instance, a response time for paypal.com should be faster than that of a phishing site. Authors in [40] investigated if DNS traffic could be correlated with other existing traffic to detect covert channel beaconing. Bilge et al. [41] explored the types of DNS features that could be used to detect malicious activity. It was found that the TTL value could occasionally create false positives due to misconfigurations from the site owners or those that were unsure of the optimal value for the DNS settings. They created a dataset of around 4.8 million observations and achieved a detection rate of 98.4%. A limitation of their approach is that the features relied on analysis over-time, causing lengthy delays. Nonetheless, it allowed them to compute averages and percentage changes on TTL values and IP addresses.

Zhauniarovich et al. [42] conducted a comprehensive review of DNS-based detection, with a focus on the practicality of detections. The factors they considered include detection latency and scalability. Similarly, the various features that are proposed in the existing literature can detect different categories of malicious behaviour, and therefore, system owners may wish to adapt their systems to detect different behaviours in-time. In summary, one can realise that a phishing detection system needs to be designed with technologies driven by modularity and horizontal scalability.

Currently, very few layered phishing detection approaches are proposed in the existing literature. These provide the opportunity to detect phishing that has the potential to bypass an initial detection sensor designed to a specific feature set. Sonowal and Kuppusamy [31] designed a layered heuristic-based detection system. The system achieved an accuracy of 92.73% overall, although a small imbalanced dataset was used. Moreover, each layer could classify if a URL was malicious or benign without consulting other layers in the system. However, this could lead to a bias classification resulting in higher Type I and Type II errors. Smadi et al. [43] combined a neural network that is used for email classification with reinforcement learning. Despite the ability to adapt to new learning environments, it would be a resource-intensive engine due to the high number of features that were used. [44] used a multi-classification method for email phishing detection. This involved forward-passing observations, e.g., if the outputs for T_1_ and T_2_ are different, they forward-pass to T_3_. Although this is similar to our approach, the technique was layered by a different algorithm for each tier, but maintained the same feature set. On the contrary, our work is focused on delivering a layered feature approach, which results in a better detection rate compared to single-layer approaches, lower resource consumption, and a more specific method of identifying if a further classification is required.

Ref. [45] proposed a layered architecture to detect specific attack types. In their work, layer one identifies the most important features per attack type, layer two is the classifier, and layer three is a softmax layer. This approach enables the system to only process the features that were identified as the most valuable during classification and thus it saves computation resources. Contrary to [45], who triage the feature space prior to classification, our paper triages at the classification layer. This can provide flexibility to what feature space could be used if they vary dramatically in computational requirements.

An empirical study by [46] used a layered process to strengthen an ensemble of classifiers by pruning, a technique to remove minor contributions to classification. Despite efforts to enhance the pruning process and achieving a higher F-measure compared to other non-pruned techniques, the dataset in use was very small. A larger dataset will likely affect the results, and thus, the performance of the layered approach is not effectively evaluated against existing and popular algorithms.

Finally, an approach by Nasr et al. [47] consisted of using an adversarial algorithm to perform inference attacks against trained models. Song et al. [48] proposed two new methods of exploiting the structural properties of adversarial conscious datasets, thus proving the investigated inference defences ineffective.

## 4. Two-Layered Phishing Detection Framework

In this section, we introduce our novel two-layered approach to detect phishing domains, which utilises both domain and DNS packet-level information to create static and dynamic features, as well as a predetermined set of upper and lower boundaries.

### 4.1. Approach

**Figure 1:**
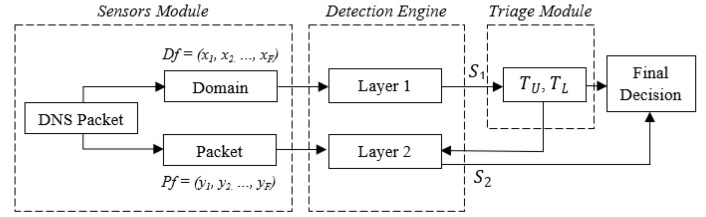
The two-layered phishing detection framework.

As depicted in Figure 1, our framework consists of three components, namely *Sensors Module*, *Detection Engine*, and *Triage Module*. The *Sensors Module* is responsible for the collection and pre-processing of the data, the *Detection Engine* includes the prediction scoring subcomponents, and the *Triage Module* compares the scoring results of *Layer 1* with the predefined thresholds. When a URL is requested by the user, the relevant DNS request and the associated returning packets are aggregated and processed by the *Sensors Module*. The processed data from the Domain (static features) is then passed to the *Layer 1* subcomponent of the *Detection Engine*, which is responsible for producing a prediction score, with the use of a supervised machine learning algorithm, that is consumed by the *Triage Module*. If the score is equal to or greater than the predefined threshold, the *Triage Module* uses it to determine the nature of the request, i.e., producing the final decision of the framework. Differently, the processed data from the Domain (dynamic features) is sent to the *Layer 2* subcomponent of the *Detection Engine*. Layer 2, in turn, produces another prediction score with the use of supervised machine learning and determines if the URL is benign or phishing, i.e., producing the final decision of the framework.

More specifically, the *Sensors Module* includes the data collection sensor(s), an essential part of the framework, on which the remaining components rely on in order to function. The sensor(s), which can be deployed as either a centralised or decentralised architecture [49] based on the requirements of the system owner’s infrastructure, capture users’ traffic and filter DNS packets. Every time a user requests to access a particular URL (e.g., via a browser or other software or tool), the initial DNS request packet becomes an input to our framework. Specifically, the *Query Name* field from the *DNS Question Record* layer is extracted and processed. The processing involves using feature engineering functions, which will be further explained in Section 4.2. The results of this process are stored in two feature vectors, namely *Df* and *Pf*. Thus, the *Sensors Module* acts as a producer for the *Detection Engine*.

The *Detection Engine* consists of two layers, each of which consists of a trained instance of a machine learning algorithm discussed in Section 2. Each layer ingests a different set of data produced by the *Sensors Module* to calculate a non-binary prediction score. The prediction score refers to the result of the machine learning algorithm, i.e., the probability of a URL being phishing. *Layer 1* consumes the data stored in the feature vector *Df* and produces the prediction score *S*_1_, while *Layer 2* consumes the data from *Pf* and produces *S*_2_. These scores represent the probability that a given URL is phishing.

The *Triage Module* is responsible for consuming the prediction score *S*_1_ and determining if the URL will be sent to *Layer 2* or not. For this reason, our framework requires that an upper and lower decision boundary, *T_U_* and *T_L_* respectively, are defined. These have to be set by the system-owner, taking into account the requirements of the infrastructure and the behaviour or profiles of the users. The decision boundaries must be chosen by the system owner so that the non-confident predictions, i.e., *S_1_*, fall within the defined boundaries, are captured by the *Triage Module,* and are sent to *Layer 2*. Therefore, if *S*_1_ ≤ *T_L_*, the URL is determined as benign, while if *S*_1_ ≥ *T_U_*, the URL is determined as phishing. As explained earlier, if *T_L_* < *S*_1_ < *T_U_*, then the URL is sent to *Layer 2*, where *S*_2_ is calculated. Finally, if *S*_2_ *< G*, where *G* is a predefined value, the URL is determined as benign, while *S*_2_ ≥ *G* is determined as phishing.

### 4.2. Feature Selection

Data arriving at the *Sensors Module* is subjected to a process of feature engineering. The features that have been chosen in this work are specific to the *DNS Question Record* and are split into two categories, namely static and dynamic, and vary with regards to their *tamper resistance*.

The features contained in the feature vector *Df* are derived from the domain name (Features No. 1–6, Table 1) and have been extensively studied and used in the literature, achieving strong detection performance [31,50,51]. As presented in [52], the lengths of malicious domain names tend to be significantly larger than the lengths of benign ones. This is likely to confuse the victims with information overload, leading to users refraining from placing the domain into question [50]. In the current threat landscape, it is common for phishing sites to be shared via email, where long links might not be visible on handheld devices, such as smartphones, due to their limited screen sizes. Similarly, the number of dots in the domain can be the result of an act of impersonation [50]. In addition, the domain’s creation date can be used to detect campaigns that leverage a real-world crisis, as [53] reported a surge in domain registrations associated with the Coronavirus outbreak in 2020. Even though an attacker can directly manipulate these features, e.g., by changing the domain length, these features are not computationally expensive and have been proven to suffice for the majority of phishing instances [31,50,51].

While some of the more advanced phishing campaigns that are carefully planned to circumvent detection may not be detected using the features of vector *Df*, the remaining features, i.e., the ones contained in *Pf* that derive from the DNS packet (Features No. 7–13, Table 1), are less likely to be tampered with by a threat actor. This holds true due to their dynamic nature, which is caused by the underlying infrastructure, and thus can be used to detect misconfigured TTL values or DNS data unavailable in nearby caches.

Here we note that the packet delta (Feature 12, Table 1) feature refers to the time difference between the two timestamps recorded in a DNS request and its corresponding response. Our experiments suggest that the packet time interval is considerably higher for phishing domains, which is easily observed by their standard deviation, *σ_Ben_* = 0.179712 and *σ_Phish_* = 89.154237, and mean values, *µ_Ben_* = 0.85355 and *µ_Phish_* = 4.533967. This makes the packet time interval a valuable feature for our *Detection Engine*, and, to the best of our knowledge, we are the first to use this feature in this domain. This holds true as in general, the domain-relevant data for phishing sites are less likely to be stored in a DNS cache, contrary to domains for benign sites.

## 5. Implementation

**Figure 2:**
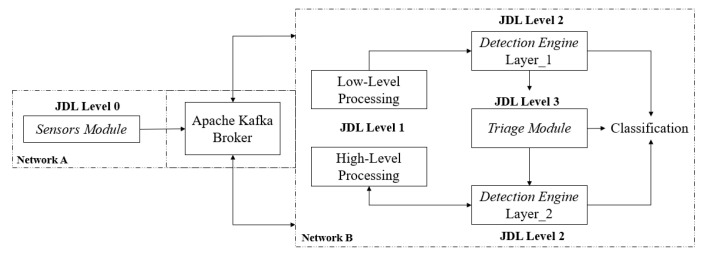
Implementation of the JDL levels relative to our framework’s components.

In this section, we present the implementation of our two-layered framework for phishing detection. Our implementation follows the JDL model to provide coherence for each of the layers of implementation. More specifically, as summarised in Figure 2, the implementation of our phishing detection framework applies the following JDL levels:

- *Level 0*—the collection of signal-level data for early pre-processing as part of the Sensor Module. Herein, the data is collected from a sensor that is deployed as an agent on user devices. Then, the necessary packet data aggregation is performed to enable further processing. This allows the preservation of user privacy by only forwarding the relevant data across the network via a data streaming technology.
- *Level 1*—Object Assessment/Refinement. In this level, which is also included in the Sensor Module, the identification and extraction of features in the data output from *Level 0* take place. Our system uses a layered approach during detection, and therefore data fusion will occur as per the response of the *Triage Module* and might include the collection of external data sources, such as WHOIS, to refine the objects’ state, i.e., the creation of a domain date.
- *Level 2*—Situation Assessment/Refinement. This includes both layers of the *Detection Engine* and involves the detection of unusual characteristics concerning objects of interest, i.e., Domain Name System features, to achieve phishing recognition.
- *Level 3*—Impact Assessment. The *Triage Module* uses the *Layer 1* classification as a feature whereby the decision boundary represents a level of reasoning. The reasoning works to capture behaviour relations to the initial object under classification as part of the fusion to create alerts.

### Experimental Setup

**Figure 3:**
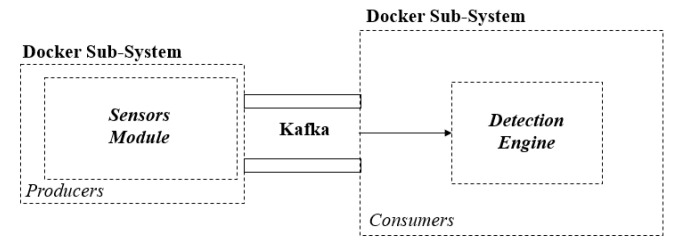
The high-level architecture of the test environment.

To demonstrate our framework, we used the test environment that is summarised in Figure 3 using an Intel Core i7-8700 at 3.2GHz, 32GB RAM. It consists of two sub-systems bridged by Kafka, namely, (i) the network sensor fleet for data collection (Sensor Module) and (ii) the detection engine (*Detection Engine* and *Triage Module*). The two sub-systems use Docker containers, which allows the separation of underlying applications. They are bridged at the virtual network layer to enable producer/consumer data streaming. The detection is the output of the system, which can be used to provide situational awareness to a decision-making entity.

We have implemented a prototype of the architecture that is summarized in Figure 2 to demonstrate how to effectively stream data live for processing in a production environment, enabling our multi-layered phishing detection framework to operate. Our implementation adheres to the need to (a) protect from single-point-of-failure (SPoF), (b) use a decentralised network sensor fleet, and (c) leverage infrastructure technologies that allow the implementation to scale.

This work considered different architectures for an intrusion detection system, namely centralised, decentralised, and distributed architecture [49]. The network sensor fleet sub-system operates in a decentralised manner to overcome the drawbacks of centralisation. These include a SPoF, limited scalability, CPU overload, and maintaining processing performance. Firstly, by decentralising the architecture, the throughput capacity of packets is increased by each machine observing the traffic in and out of the network interface. Secondly, the User Datagram Protocol (UDP) packets are captured and filtered by DNS, which reduces the resources required for data collection. Moreover, Docker was selected for performance reasons as it allows us to install and run isolated instances of technologies without the need to fully virtualise at the kernel level.

**Figure 4:**
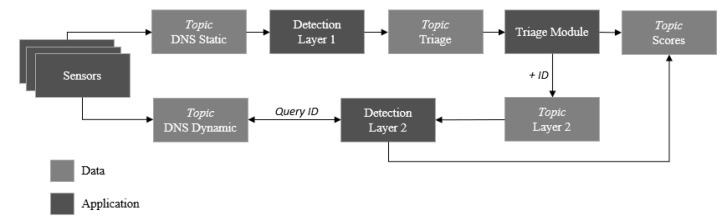
The data flow diagram for the test environment.

A core module that interfaces to the sensor fleet to collect observations is Apache Kafka (see Figure 3), a low latency data-streaming technology. The technology is renowned for its development and use by companies such as LinkedIn, Twitter, Spotify, and Uber. Kafka was selected to ingest and move large amounts of data with high performance, which is achieved by using a publish-subscribe messaging principle. Kafka provides to the detection system the ability to horizontally scale with minimal data retention and topic group consumption. In-sync replicas enable the system to stay alive if a Kafka broker goes offline. The data flow for live-streaming the test environment is depicted in Figure 4.

## 6. Evaluation

### 6.1. Data Collection

To demonstrate our approach, we used multiple data sources to reduce the detection engine’s sensitivity to new data. In this regard, our dataset had to include live domains; therefore, sources of threat intelligence that are updated daily were necessary. To this end, in July 2020, we created a dataset that included 18,030 benign domains, which were collected from Alexa, (https://www.alexa.com) as well as 18,030 unique and active phishing domains, where 14,504 phishing domains were collected from PhishTank (https://www.phishtank.com) and the rest from OpenPhish (https://openphish.com).

To train and evaluate the performance of the selected machine learning algorithms with the aforementioned features, the dataset was enriched initially by confirming an active status through visiting each domain. This reduced the volume to 17,244 benign and 7970 phishing domains using the Google DNS service. Furthermore, the data enrichment process of refining the object (domain) state reduced the dataset further to 16,494 benign and 7053 phishing domains.

Existing literature often uses balanced datasets for training and testing, as seen in [54,55]. However, Das et al. [12] state the concern that using a balanced test set does not reflect the real world. This holds true because real network traffic includes proportionally more benign instances than phishing ones. For this reason, our work used a balanced dataset only during the training of the classifiers to prevent introducing bias (see Table 2). During their testing we used an imbalanced dataset, reflecting conditions similar to a real production system.

Finally, here we choose to set *G* = 0.5, which is the most commonly used threshold for classification algorithms. This means that when the prediction score *S*_2_ equals 0.5, we choose to classify the corresponding URL as phishing. As a result, *Layer 2* might misclassify a benign site as a phishing site, and thus increase the FP. However, this work accepts this trade-off as, alternatively, an FN might occur, which poses a greater security risk.

### 6.2. Results

In this section, we examine the *Detection Engine’s* performance with the use of the aforementioned dataset. Initially, we evaluate the performance of the feature sets presented in Section 4.2 and then focus on the performance of the proposed layered architecture. To evaluate the performance of the feature sets, we use metrics that are commonly used to measure the performance of data modelling in intrusion detection [49], namely: (i) Precision, (ii) Recall, (iii) F1 score, (iv) Accuracy, and (v) Matthews correlation coefficient (MCC).

(1) $$Precision=\frac{TP}{TP+FP}$$

(2) $$Recall=\frac{TP}{TP+FN}$$

(3) $$F1=2\times \frac{Precision\times Recall}{Precision+Recall}$$

(4) $$Accuracy=\frac{TP+TN}{TP+TN+FN+FP}$$

(5) $$MCC=\frac{TP\times TN-FP\times FN}{\sqrt{\left(TP+FP\right)\left(TP+FN\right)\left(TN+FP\right)\left(TN+FN\right)}}$$

Most of the existing literature on phishing detection uses a single-model approach, where several features are used to detect phishing attacks. On the contrary, this work proposes a layered approach that is evaluated to identify the effectiveness of triaging the initial classification confidence. The classifiers that are evaluated herein, which have been described in Section 2, are: Multilayer perceptron, Support Vector Machine, Naïve Bayes, and Decision Trees (see Table 3).

With regards to the demonstration of our proposed framework, features 1– 6 from Table 1 were used as the feature vector *Df* for *Layer 1*. These are static and, thus, can be immediately calculated from the domain. Similarly, features 7–13 were used in *Pf* for *Layer 2*. This was deemed appropriate for refining the object state (domain), as contacting external data sources (such as WHOIS) and tracking returning packets is dependent on collecting the dynamic features 7–13.

Initially, we evaluate each classifier’s performance as one layer against three different feature sets. Thus, for each classifier in scope (refer to Table 3), we study three different cases using features (i) F1–F6, (ii) F7–F13, and (ii) F1–F13. We then evaluate the effectiveness of layering the features, such that two separate model instances, i.e., one for *Layer 1* and one for *Layer 2*, are trained on the layered feature sets as presented in Section 3.

In all the cases where a classifier uses all the available features, it achieves the best detection scores (see Table 3). Our results show in Table 3 that the MLP and SVM classifiers using features F1–F13 outperform the rest of the classifiers and feature set combinations we examined, with respect to the achieved accuracy (89%) and MCC (75%). MLP using features F7-13 seems to outperform SVM using features F7-13 in terms of accuracy (81% and 40%, respectively) and MCC (59% and 15%, respectively). We did not notice any major difference in the results for features F1–F6. Despite MLP and SVM having a weaker precision than Naïve Bayes in some instances, as summarised in Table 3, they achieve a balance between precision and recall, which is crucial for phishing detection. This holds true as it provides benefits to the end-user, i.e., by reducing misclassifications. In this regard, MLP outperforms the other classifiers in the F1-Score, thus balancing precision and recall and being a stronger classifier overall.

We now evaluate the proposed framework against a range of values for the decision boundaries *T_U_* and *T_L_*. Here we choose to assign symmetrical values for the boundaries. However, optimal boundary values can be acquired through experimentation and tuning, as will be discussed further in the next section.

**Figure 5:**
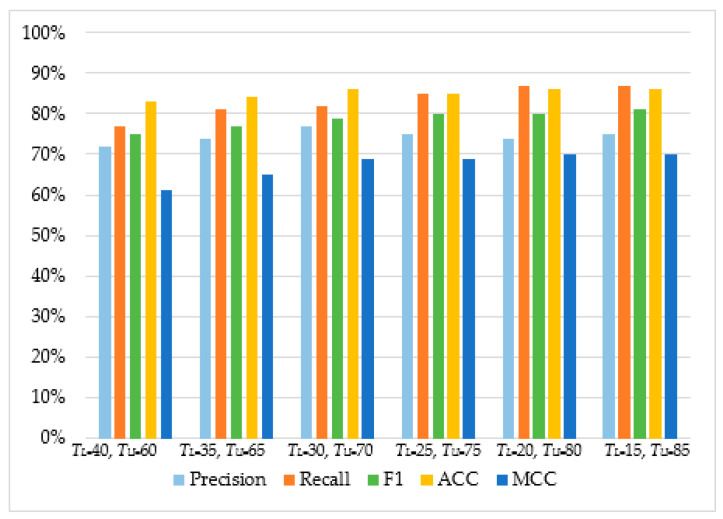
The performance of Multilayer Perceptron.

**Figure 6:**
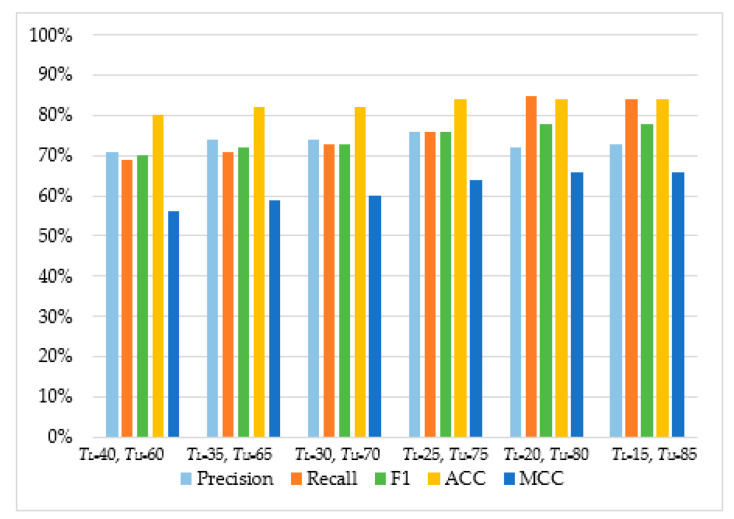
The performance of Support Vector Machine.

**Figure 7:**
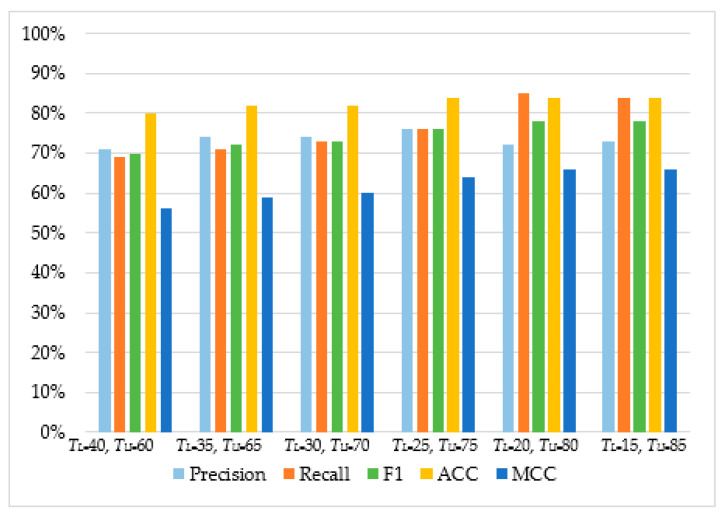
The performance of Naïve Bayes.

**Figure 8:**
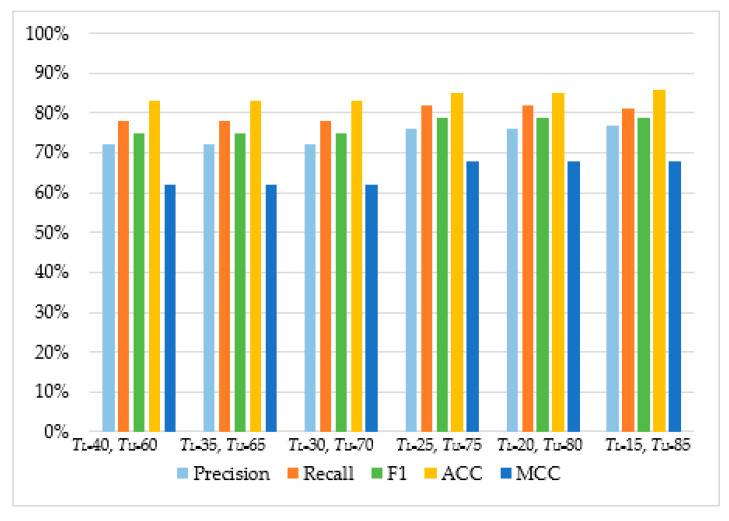
The performance of Decision Tree.

As can be observed in Table 4 and Figure 5, Figure 6, Figure 7 and Figure 8, in most cases, using *T_U_* = 15 and *T_L_* = 85 achieves the best classification scores. This is expected as we are allowing a higher range between the two boundaries and, thus, a higher number of requests are passed through *Layer 2*, where they get classified using the second set of features. Moreover, the results suggest that Naive Bayes and Decision Trees when used with a layered approach outperform their single-layer counterparts that use all the available features. MLP had the highest score, with Decision Tree having the second-highest due to a small deficit in the F1 and MCC metrics. When comparing the two-layered MLP with the single-layered MLP with all the available features, one can see that the former is performing similarly to the latter with only 3–5% deficit in F1, Accuracy, and MCC scores.

## 7. Discussion and Conclusions

In the current threat landscape, phishing constitutes a significant risk for web users. In this paper, we propose and demonstrate our novel two-layered detection framework to detect phishing domains. Based on our evaluation with a representative dataset, which contains real and active phishing instances, we demonstrate that our detection system achieves detection scores that are comparable with the state of the art. Nonetheless, our results suggest that organisations can sacrifice 3–5% in detection scores, such as F1, accuracy, and MCC, but utilise a system that is better suited for production environments. This holds true, as our proposed framework places classification operations that are resource-demanding in the second layer, thus achieving better system performance. It is worth noting that the focus of this work was not to find and tune the optimum feature set for each detection layer. Instead, we explored and proved the feasibility of using a multi-layered approach which would avoid resource consumption with the aid of the *Triage Module*.

The literature has covered different strategies that threat actors use in the current landscape to (*a*) masquerade as an existing benign site and (*b*) bypass detection (e.g., with poisoning attacks). Past literature mostly relies on the use of the domain name as a feature, which can be easily manipulated by the threat actors. While our detection system relies on the domain as a classification feature, it provides additional tamper resistance by using a different set of features in the second layer that cannot be easily manipulated by threat actors. Furthermore, our system avoids the use of URLs as a classification feature, contrary to previous works as well as many content-based solutions. This is to adhere to recent developments on the web where the fact that TLS/SSL has become ubiquitous, even amongst phishing sites, can prevent the use of URL and content-based features. Our framework collects the domain name from the *Query Name* in DNS packets, which currently remain unencrypted despite the use of TLS/SSL.

A key component of our framework is the *Triage Module*, which is based on a set of decision boundaries and has the objective of capturing the observations that are least confident in the initial detection. The decision boundaries are a system parameter that must be set by the system owner in a way that the non-confident predictions are reclassified, based on past incidents, following data modelling or based on the requirements of the infrastructure and the behaviour or profiles of the users. While in our evaluation we have used static values for the decision boundaries, we consider that these values would need to be regularly updated in a production environment. Even though this falls outside the scope of this work, we consider that in a production environment, the decision boundaries must be updated dynamically through an automated module or by the system owner following a data-driven exercise to enable non-confident predictions from *Layer 1*, i.e., *S*_1_ falls within the defined boundaries, to be captured by the *Triage Module* and sent to *Layer 2*.

It is worth noting that, as our results suggest, the decision boundaries must be wisely selected, otherwise correctly classified observations (TP and TN) from *Layer 1* risk being misclassified in *Layer 2*, which creates additional FP and FN. The best-case scenario of this selection would be the use of a decision boundary that would send all or almost all FN and FP to *Layer 2* while passing minimum or no TP and TN. However, this best-case scenario could be unachievable within a production environment, as it would assume that all the misclassifications are always identified and forwarded to the next layer. We leave this study as future work.

We have implemented a prototype of the architecture to demonstrate how to effectively stream data live for processing in a production environment enabling our multi-layered phishing detection framework to operate. To the best of our knowledge, this is the first application of this technique in such a domain which is in a production-ready state. By demonstrating the architecture in alignment with the JDL model, the viability for adoption in an organisation expands, as the JDL process and modularity encourages development in other layers of JDL, namely, Situation Refinement, Process Refinement, and Mission Refinement.

For future work, we plan to include a sophisticated data fusion process as part of JDL level 2 (Situation Refinement). Furthermore, we plan to explore if a larger dataset would help us improve the detection scores of our system.

## Figures and Tables

**Table 1:** The features used in the *Detection Engine*.

#	Feature	Type
1	Domain Length	*Static*
2	SLD Length	*Static*
3	TLD Length	*Static*
4	TLD	*Static*
5	Number of dots in Domain Name	*Static*
6	Domain number to character ratio	*Static*
7	Domain Creation Date	*Static*
8	Registrar Name	*Static*
9	Length of Response	*Dynamic*
10	Count of Resource Requests	*Dynamic*
11	Count of Resource Responses	*Dynamic*
12	Packet Delta	*Dynamic*
13	Time to Live (TTL) of the Resource Record	*Dynamic*

**Table 2:** The data used in the experiments.

	Benign	Phishing	Total
**Train**	4937	4937	9874
**Test**	2116	1058	3174
**Total**	7053	5995	13,048

**Table 3:** Classifier performance on a single-layered model.

Algorithm	Features	Precision	Recall	F1-Score	ACC	MCC
Multilayer Perceptron	F1–F6	65%	71%	68%	78%	51%
	F7–F13	69%	78%	74%	81%	59%
	F1–F13	**83%**	**84%**	**84%**	**89%**	**75%**
Support Vector Machine	F1–F6	70%	65%	67%	79%	52%
	F7–F13	36%	97%	52%	40%	15%
	F1–F13	**84%**	**82%**	**83%**	**89%**	**75%**
Naïve Bayes	F1–F6	62%	61%	61%	75%	42%
	F7–F13	35%	98%	52%	39%	13%
	F1–F13	88%	40%	55%	78%	49%
Decision Trees	F1–F6	73%	60%	66%	79%	52%
	F7–F13	72%	60%	66%	79%	51%
	F1–F13	72%	82%	77%	83%	64%

**Table 4:** Classifier performance on a two-layered architecture.

Algorithm	Decision Boundary	Precision	Recall	F1	ACC	MCC
Multilayer Perceptron	*T_L_* = 40, *T_U_* = 60	72%	77%	75%	83%	61%
	*T_L_* = 35, *T_U_* = 65	74%	81%	77%	84%	65%
	*T_L_* = 30, *T_U_* = 70	77%	82%	79%	86%	69%
	*T_L_* = 25, *T_U_* = 75	75%	85%	80%	85%	69%
	*T_L_* = 20, *T_U_* = 80	74%	87%	80%	86%	70%
	*T_L_* = 15, *T_U_* = 85	75%	87%	**81%**	**86%**	**70%**
Support Vector Machine	*T_L_* = 40, *T_U_* = 60	71%	69%	70%	80%	56%
	*T_L_* = 35, *T_U_* = 65	74%	71%	72%	82%	59%
	*T_L_* = 30, *T_U_* = 70	74%	73%	73%	82%	60%
	*T_L_* = 25, *T_U_* = 75	76%	76%	76%	84%	64%
	*T_L_* = 20, *T_U_* = 80	72%	85%	**78%**	**84%**	**66%**
	*T_L_* = 15, *T_U_* = 85	73%	84%	**78%**	**84%**	**66%**
Naïve Bayes	*T_L_* = 40, *T_U_* = 60	66%	58%	61%	76%	44%
	*T_L_* = 35, *T_U_* = 65	70%	56%	62%	77%	47%
	*T_L_* = 30, *T_U_* = 70	73%	56%	**64%**	79%	50%
	*T_L_* = 25, *T_U_* = 75	74%	55%	63%	79%	49%
	*T_L_* = 20, *T_U_* = 80	78%	52%	62%	79%	51%
	*T_L_* = 15, *T_U_* = 85	79%	51%	62%	**79%**	**51%**
Decision Tree	*T_L_* = 40, *T_U_* = 60	72%	78%	75%	83%	62%
	*T_L_* = 35, *T_U_* = 65	72%	78%	75%	83%	62%
	*T_L_* = 30, *T_U_* = 70	72%	78%	75%	83%	62%
	*T_L_* = 25, *T_U_* = 75	76%	82%	79%	85%	68%
	*T_L_* = 20, *T_U_* = 80	76%	82%	79%	85%	68%
	*T_L_* = 15, *T_U_* = 85	77%	81%	**79%**	**86%**	**68%**
